# U-SMAS: ultrasound findings of the superficial musculoaponeurotic system

**DOI:** 10.1590/0100-3984.2024.0035

**Published:** 2024-09-04

**Authors:** Luciana C. Zattar, Gladstone Faria, Ricardo Boggio

**Affiliations:** 1 Hospital Sírio-Libanês, São Paulo, SP, Brazil; 2 Instituto Boggio, São Paulo, SP, Brazil

**Keywords:** Superficial musculoaponeurotic system, Dermatology, Ultrasonography/methods, Skin/anatomy & histology, Rejuvenation, Cosmetic techniques, Sistema musculoaponeurótico superficial, Dermatologia, Ultrassonografia/métodos, Pele/anatomia & histologia, Rejuvenescimento, Técnicas cosméticas

## Abstract

The superficial musculoaponeurotic system (SMAS) is a complex fibrous network connecting facial muscles to the dermis, with varying morphological characteristics across different facial regions. Recent studies have identified five distinct types of SMAS morphology, highlighting the need for region-specific interventions in facial rejuvenation. This pictorial essay explores ultrasound imaging of the SMAS using ultra-high frequency (24–33 MHz) probes, known as U-SMAS. Analysis of 186 full-face U-SMAS scans revealed consistent patterns in the facial and neck layers, with regional variations aligning with the Sandulescu classifications: type I (preparotideal); type II (chin and lip); type III (eyelid); type IV (temporal and parotideal); and type V (cervical). Understanding these morphological differences is crucial for accurate interpretation of ultrasound images and for optimizing pre-procedural assessments to ensure that aesthetic treatments are safe and effective. Knowledge of the SMAS architecture enhances the ability to visualize facial and neck anatomy accurately, particularly through U-SMAS imaging, ensuring comprehensive patient care in rejuvenation procedures.

## Abstract (pt)

O sistema musculoaponeurótico superficial (SMAS) é uma rede fibrosa complexa que conecta os músculos faciais à derme, com características morfológicas variadas em diferentes regiões faciais. Estudos recentes identificaram cinco tipos distintos de morfologia do SMAS, destacando a necessidade de intervenções específicas em cada região para a rejuvenescimento facial. Este ensaio iconográfico explora a imagem por ultrassom do SMAS usando transdutores de ultra-alta frequência (24-33 MHz), conhecidas como U-SMAS. A análise de 186 exames de ultrassom de rosto completo revelou padrões consistentes nas camadas faciais e do pescoço, com variações regionais alinhadas com as classificações de Sandulescu: tipo I (preparotideal), tipo II (queixo e lábio), tipo III (pálpebra), tipo IV (temporal e parotideal) e tipo V (cervical). Compreender essas diferenças morfológicas é crucial para uma interpretação precisa do ultrassom e para otimizar avaliações pré-procedimento para tratamentos estéticos seguros e eficazes. O conhecimento da arquitetura do SMAS melhora a capacidade de visualizar com precisão a anatomia facial e do pescoço, especialmente por meio de imagens de ultrassom, garantindo cuidados abrangentes ao paciente em procedimentos de rejuvenescimento.

## INTRODUCTION

**Figure 1:**
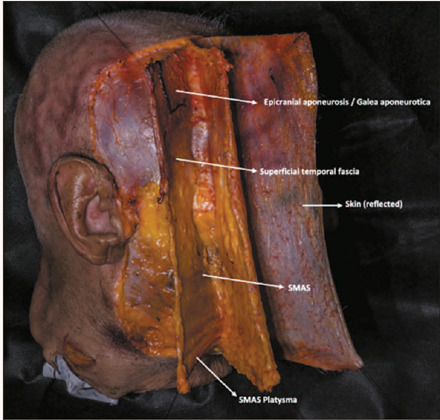
Fresh-frozen specimen photography showing the SMAS as a continuous, organized fibrous network that connects the facial muscles with the dermis. (Photograph by Thalita Melo of ExtraCut Global, Cascais, Portugal).

**Figure 2:**
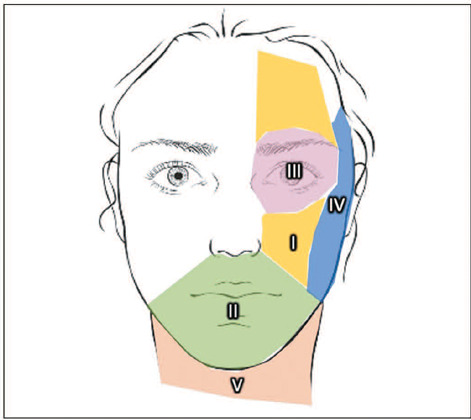
Schematic representation of the five different SMAS types, as recently described: type I: preparotideal region, lateral to the nasolabial fold; type II: chin and lip region, medial to the nasolabial fold; type III: lower and upper eyelid region: type IV: temporal and parotideal region: type V: cervical region, comprising the septum fibrosus profundus and septum fibrosus.

The superficial musculoaponeurotic system (SMAS) is defined as an organized continuous fibrous network that connects the facial muscles to the dermis (Figure 1). It comprises fat cells, collagen, and elastic fibers, forming a three-dimensional framework extending from the galea aponeurotica to the platysma muscle^(1,2,3)^. The concept of the SMAS, first described and named by Mitz & Peyronie in 1976^(4)^, has been the subject of (occasionally contentious) debate in the literature^(5,6,7)^. Five distinct morphological types of SMAS have recently been described, demonstrating specific morphology in different facial areas (Figure 2). Therefore, region-specific aesthetic and surgical approaches may be necessary for facial rejuvenation^(5,6,7,8,9,10)^. It is important to respect the layered arrangement of the facial soft tissue in order to achieve better results and optimal outcomes. For example, injecting filler results in a stretching effect in SMAS type IV whereas it volumizes in SMAS type I^(11)^.

In the past decade, there has been significant focus on the intricate layered anatomy of the face, which is among the most complex areas of the human body. This attention is due to the increasing number and variety of facial rejuvenation procedures being performed^(5,10,12,13,14,15)^. Despite the importance of the SMAS in facial rejuvenation^(12,16,17)^, there is as yet no clear anatomic definition with imaging findings and description of the SMAS. Some authors have attempted to analyze the appearance of the SMAS on computed tomography (CT) and magnetic resonance imaging scans of the face^(1,18)^. However, ultrasound would be more well suited to this characterization because it provides optimal anatomical information of the skin and allows the facial layers to be differentiated^(19)^.

This pictorial essay aims to illustrate and describe ultrasound findings of the SMAS obtained with ultra-high frequency (24–33 MHz) probes, known as U-SMAS. To that end, images from 186 full-face U-SMAS examinations were analyzed in an online archive. All of the ultrasound images were obtained by a qualified radiologist with a high-resolution system (Aplio i700; Canon Medical Systems, Otawara, Japan) in B-mode, including superb microvascular imaging Doppler and elastography with ultra-high-frequency probes (24–33 MHz), identifying regional differences and characteristics of all five SMAS types, as well as comparing them with the descriptions established by Sandulescu^(6,7,8,9)^. Although informed consent is not required for this type of study, all patients gave written informed consent, as required in the examination protocol and in accordance with the Declaration of Helsinki.

**Figure 3:**
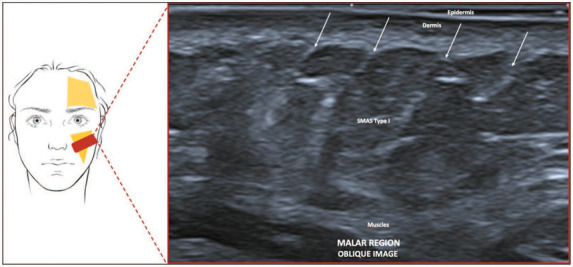
SMAS type I. Schematic representation of the examination and U-SMAS B-mode 24 MHz ultrasound image of the left malar region, showing a pattern of a hypoechoic subcutaneous tissue and hyperechoic vertical septa (arrows).

In all cases, we found consistency in the U-SMAS imaging patterns of the facial and neck layers. Regional differences in the imaging aspects of the fibrous septa and subcutaneous fat tissue were observed, in keeping with the findings of Sandulescu^(2,6,7,8,9,20)^. We defined the SMAS types as follows:

**Type I—Preparotideal region, lateral to the nasolabial fold.** In all cases, we found a pattern of a hypoechoic subcutaneous tissue and hyperechoic vertical septa (Figure 3), similar to the hypodermis in other regions of the body.

**Figure 4:**
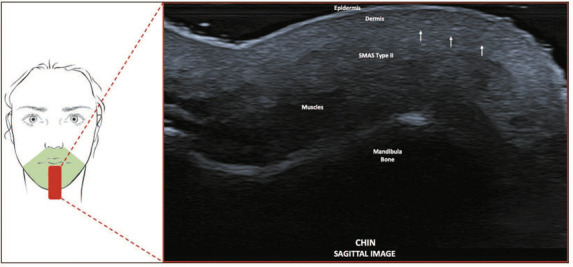
SMAS type II. Schematic representation of the examination and U-SMAS B-mode 24 MHz ultrasound image of the chin, showing that the subcutaneous tissue has a heterogeneous, hyperechoic aspect, with less differentiation between the dermal and hypodermal layers (arrows).

**Type II—Chin and lip region, medial to the nasolabial fold.** We noted a heterogeneous, hyperechoic aspect to the subcutaneous tissue in all ultrasound images (Figure 4), with less differentiation between the dermal and hypodermal layers. In this region, compression may help to analyze the deeper structures and layers.

**Figure 5:**
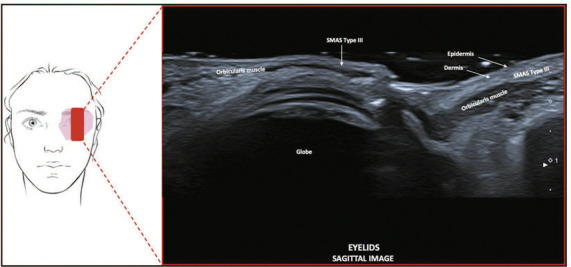
SMAS type III. Schematic representation of the examination and U-SMAS B-mode 24 MHz ultrasound image of the eyelid region, showing a thin, fat-poor hyperechoic layer between the skin and the orbicularis muscle.

**Type III—Lower and upper eyelid region.** On ultrasound images, this appears as a thin, fat-poor, hyperechoic layer between the skin and the orbicularis muscle (Figure 5).

**Figure 6:**
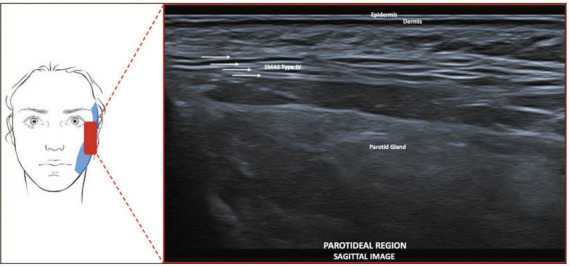
SMAS type IV. Schematic representation of the examination and U-SMAS B-mode 24 MHz ultrasound image of the parotideal region, showing hyperechoic horizontal lines, parallel to the skin (arrows).

**Type IV—Temporal and parotideal region.** On ultrasound, it shows hyperechoic horizontal lines, parallel to the skin (Figure 6).

**Figure 7:**
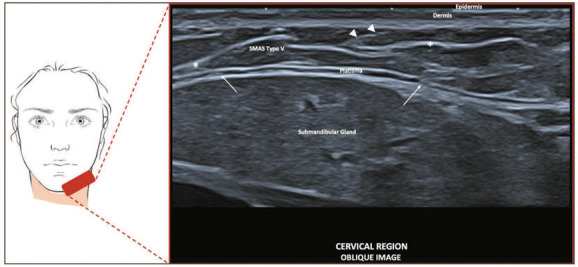
SMAS type V. Schematic representation of the examination and U-SMAS B-mode 24 MHz ultrasound image of the cervical region, showing hypoechoic subcutaneous tissue with a parallel fibrous septum (asterisk) and a vertically aligned septum (arrowheads) connecting the skin to the platysma muscle, as well as a small fibrous septum within the muscle (arrows).

**Type V—Cervical region.** We found hypoechoic subcutaneous tissue with a parallel, fibrous, vertically aligned septum connecting the skin to the platysma muscle, as well as a small fibrous septum within the muscle (Figure 7).

## DISCUSSION

The SMAS serves to link the facial muscles to the skin, enabling facial mimicry, and shows regional differences in architectural morphology. In recent years, studies have put forward various classifications of SMAS architecture concerning how it interacts with the muscles involved in facial mimicry and with dynamic aging processes like the formation of folds and creases. These classifications are based on the arrangement of fibrous septa and regional variations, and they can also vary between genders.

The absence of a clear understanding of the SMAS and of a distinction between it and the fat compartments of the face and neck has led to diverse interpretations and debates^(2,3,4,5,6,7,8,9,10,20,21,22)^. According to the most recent studies, the SMAS can be divided into types by configuration, and in each region, we also have noticed different imaging patterns and findings, as follows:

**Type I—Preparotideal region, lateral to the nasolabial fold.** This area contains vertically oriented fibrous septa that connect to the skin parallel to the muscle planes and at a right angle to the skin. These septa envelop individual bundles of mimic muscles. On ultrasound, this configuration appears as hypoechoic subcutaneous tissue and hyperechoic vertical septa, similar to the hypodermis in other regions of the body.

**Type II—Chin and lip region, medial to the nasolabial fold.** This area is composed of a dense, irregular network of fibers with sparse adipose cushions and fibromuscular septa that are thicker and more densely distributed than in the other facial regions. It connects the bundles of the orbicularis oris muscle to the perioral dermis, which gives a heterogeneous and hyperechoic aspect to the subcutaneous tissue on ultrasound images.

**Type III—Lower and upper eyelid region.** In this region, the connective tissue is fat-poor and is arranged in a loose, irregular fibroelastic network. That network is composed of a fibrous mesh that connects to the orbicularis oculi muscle above the dermis and below the subcutaneous tissue that covers the lids. On ultrasound, this configuration appears as a thin, hyperechoic layer because the fibrous meshwork is poor in fat.

**Type IV—Temporal and parotideal region.** In this region, fibrous septa align parallel to the skin, as a result of the absence of facial mimicry muscles, anchoring the parotid fascia. On ultrasound, it appears as hyperechoic horizontal lines. Type I and IV SMAS tissues border the subcutaneous septum in the parotid-masseteric fascia.

**Type V—Cervical region.** This area comprises the deep fibrous septum, superficial fibrous septum, and commissural fibrotic septa, which interact with the platysma muscle and skin.

On ultrasound, the soft tissue layers can be distinguished and individualized. The normal skin is characterized by a bilaminar structure with a hyperechoic superficial line and a less hyperechoic band, which correspond to the epidermis and the dermis, respectively. The subcutaneous tissue (hypodermis) appears as a hypoechoic layer with hyperechoic fibrous septa, and the muscles have a hypoechoic fibrillar appearance with hyperechoic tendons and sheaths/fascias. These normal appearances of the structures are different in the face and neck regions, which must be because of the previously mentioned unique organizations of the SMAS.

**Figure 8:**
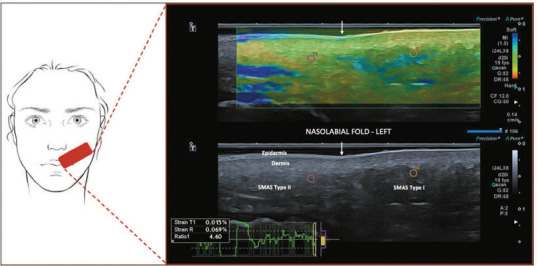
Schematic representation of the examination and elastography of the SMAS. The different configurations of the SMAS may be characterized around the nasolabial fold (arrows) by stiffer tissue in the perioral area (red: T1 marker) than in the malar area (blue: R marker), with a strain ratio of 4.6.

Elastography, a form of ultrasonography suitable for quantifying tissue strain, can also be used in the characterization of the SMAS. It provides information on tissue stiffness, independent blood perfusion, and acoustic impedance. Elastography relies on the concept that tissues vary in elasticity, allowing for differentiation between them. The strain of normal skin is known to vary across its layers. Normal skin demonstrates varying strain across its layers, with the dermis being less elastic than subcutaneous tissue. Hypodermis is not homogeneous, because of the presence of high-strain connective tissue septa and lower-strain fat tissue lobes, nerve fibers, and blood vessels^(23,24,25,26,27)^. The different configurations of the SMAS around the nasolabial fold can be characterized by stiffer tissue in the perioral area than in the malar area (Figure 8).

**Figure 9:**
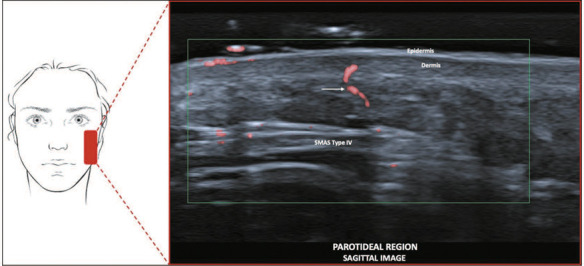
Schematic representation of the examination and Doppler superb microvascular imaging 24 MHz image, showing a corkscrew-like vessel (arrow).

In the ultrasound evaluation, SMAS-related differences in blood supply may be noted, although the micro-circulation of the SMAS remains relatively unknown and understudied. Studies show that it is nourished by two horizontally aligned vascular networks—the epimuscular and subcutaneous vascular frameworks—connected by corkscrew-like vessels (Figure 9). These findings highlight the multifunctional nature of the SMAS, which plays physical as well as immunological roles^(7)^.

**Figure 10:**
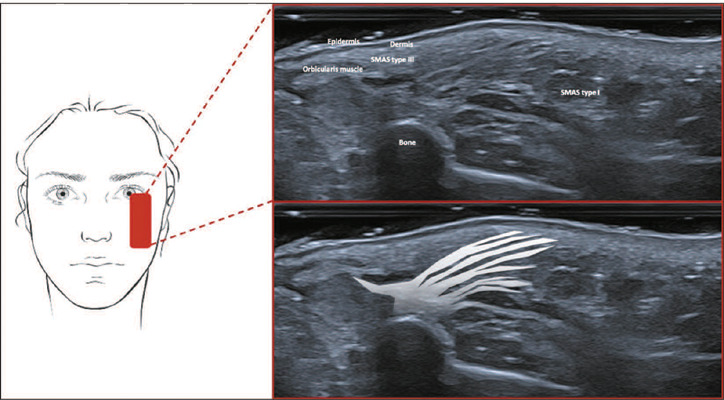
Schematic representation of the examination and U-SMAS B-mode 24 MHz ultrasound image in the zygomatic region, showing a hyperechoic structure represented in the image below, recognized as a true facial ligament, comprising the orbicularis retaining and zygomatic ligaments.

**Figure 11:**
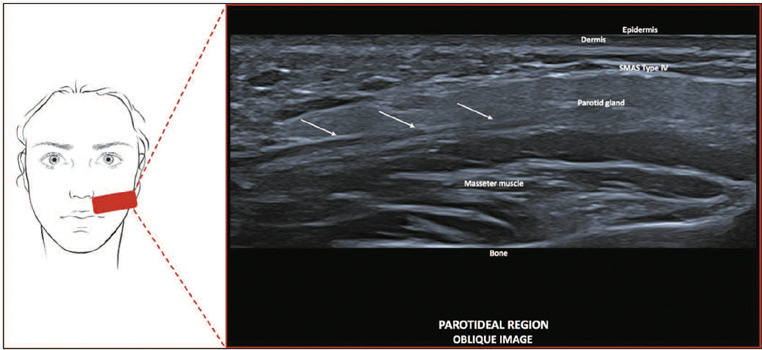
Schematic representation of the examination and U-SMAS B-mode 24 MHz ultrasound image of the parotideal region, showing a branch of the facial nerve (arrows).

Knowledge of the SMAS is also related to ligament formation and nerve localization. Fibrous connections anchor the SMAS to the skin and to the deep fascia. In certain areas, where these connections are dense, providing strong fixation points, suspension, or pathways for arterial blood nourishment, they are referred to as ligaments. Major branches of the facial nerve lie beneath the facial mimicry musculature and transverse fibrous connective tissues linking the parotid-masseteric fascia to the SMAS. Those structures enclose surgical access spaces utilized in facelift procedures^(7,13,15,17,28,29)^. True ligaments and nerves can also be studied and visualized on ultrasound (Figures 10 and 11).

These imaging findings regarding the differences among SMAS types, including flow studies and elastography, should be explored further.

## CONCLUSION

Through this pictorial essay, we have illustrated and described the ultrasound characteristics of the SMAS. Knowledge of the regional and layered facial and neck anatomy and its normal appearance on imaging examinations, especially U-SMAS, is crucial for performing optimal pre- and post-procedural analyses. A thorough understanding of this anatomy and of the differences described in this essay is essential, whereas comprehensive knowledge of the regional variation within the face and neck regions is important for the execution of any aesthetic procedure.

## References

[1] Okuda I, Abe K, Yoshioka N (2023). Objective analysis of age-related changes in the superficial
musculoaponeurotic system in Japanese females using computed
tomography. Aesthet Surg J Open Forum.

[2] Ghassemi A, Prescher A, Riediger D (2003). Anatomy of the SMAS revisited. Aesthetic Plast Surg.

[3] Whitney ZB, Jain M, Zito PM (2024). StatPearls [Internet].

[4] Mitz V, Peyronie M (1976). The superficial musculo-aponeurotic system (SMAS) in the parotid
and cheek area. Plast Reconstr Surg.

[5] Hwang K, Choi JH (2018). Superficial fascia in the cheek and the superficial
musculoaponeurotic system. J Craniofac Surg.

[6] Sandulescu T, Spilker L, Rauscher D (2018). Morphological analysis and three-dimensional reconstruction of
the SMAS surrounding the nasolabial fold. Ann Anat.

[7] Sandulescu T, Buechner H, Rauscher D (2019). Histological, SEM and three-dimensional analysis of the midfacial
SMAS – new morphological insights. Ann Anat.

[8] Sandulescu T, Blaurock-Sandulescu T, Buechner H (2018). Three-dimensional reconstruction of the suborbicularis oculi fat
and the infraorbital soft tissue. JPRAS Open.

[9] Sandulescu T, Franzmann M, Jast J (2019). Facial fold and crease development: a new morphological approach
and classification. Clin Anat.

[10] Broughton M, Fyfe GM (2013). The superficial musculoaponeurotic system of the face: a model
explored. Anat Res Int.

[11] Casabona G, Frank K, Koban KC (2019). Lifting vs volumizing – the difference in facial minimally
invasive procedures when respecting the line of ligaments. J Cosmet Dermatol.

[12] Kapoor KM, Saputra DI, Porter CE (2021). Treating aging changes of facial anatomical layers with
hyaluronic acid fillers. Clin Cosmet Investig Dermatol.

[13] Mendelson BC, Jacobson SR (2008). Surgical anatomy of the midcheek: facial layers, spaces, and the
midcheek segments. Clin Plast Surg.

[14] Ingallina F, Alfertshofer MG, Schelke L (2022). The fascias of the forehead and temple aligned—an anatomic
narrative review. Facial Plast Surg Clin North Am.

[15] Cotofana S, Lachman N (2019). Anatomy of the facial fat compartments and their relevance in
aesthetic surgery. J Dtsch Dermatol Ges.

[16] Corduff N (2021). Neuromodulating the SMAS for natural dynamic
results. Plast Reconstr Surg Glob Open.

[17] Alghoul M, Codner MA (2013). Retaining ligaments of the face: review of anatomy and clinical
applications. Aesthet Surg J.

[18] Macchi V, Tiengo C, Porzionato A (2007). Anatomo-radiological study of the superficial musculo-aponeurotic
system of the face. Ital J Anat Embryol.

[19] Zattar L, Gebrim ES, Zattar L, Cerri GG (2021). Ultrassongrafia dermatológica.

[20] Sandulescu T, Stoltenberg F, Buechner H (2020). Platysma and the cervical superficial musculoaponeurotic system –
comparative analysis of facial crease and platysmal band
development. Ann Anat.

[21] Custódio ALN, Lopes ADL, Figueiredo FC (2021). SMAS e ligamentos da face – revisão
anatômica. Aesthetic Orofacial Science.

[22] Hutto JR, Vattoth S (2015). A practical review of the muscles of facial mimicry with special
emphasis on the superficial musculoaponeurotic system. AJR Am J Roentgenol.

[23] Kleinerman R, Whang TB, Bard RL (2012). Ultrasound in dermatology: principles and
applications. J Am Acad Dermatol.

[24] Ambroziak M, Pietruski P, Noszczyk B (2019). Ultrasonographic elastography in the evaluation of normal and
pathological skin — a review. Postepy Dermatol Alergol.

[25] Xiang X, Yan F, Yang Y (2017). Quantitative assessment of healthy skin elasticity: reliability
and feasibility of shear wave elastography. Ultrasound Med Biol.

[26] Ambroziak M, Noszczyk B, Pietruski P (2019). Elastography reference values of facial skin
elasticity. Postepy Dermatol Alergol.

[27] Alfageme Roldán F (2016). Elastography in dermatology. Actas Dermosifiliogr.

[28] Sykes JM, Riedler KL, Cotofana S (2020). Superficial and deep facial anatomy and its implications for
rhytidectomy. Facial Plast Surg Clin North Am.

[29] Mendelson BC (2013). Anatomic study of the retaining ligaments of the face and
applications for facial rejuvenation. Aesthetic Plast Surg.

